# Effects of Roasting on Antibacterial and Antioxidant Properties of *Sophora japonica* Buds—The Involvements of Rutin and Quercetin Constituents

**DOI:** 10.3390/plants13233337

**Published:** 2024-11-28

**Authors:** Hieu Chi Nguyen, Hang Thi Thuy Hoang, Atsushi Miyamoto, Tien Dung Nguyen, Ha Thi Thanh Nguyen

**Affiliations:** 1 Laboratory of Pharmacology and Drug Development, Center of Excellent Research and Innovation, Vietnam National University of Agriculture, Gia Lam District, Hanoi 131000, Vietnam; nguyenhieu210.nicvb@gmail.com (H.C.N.); 32151050@sv.vnua.edu.vn (H.T.T.H.); 2 National Institute for Control of Vaccines and Biologicals, Hoang Mai District, Hanoi 128100, Vietnam; 3 Department of Veterinary Pharmacology, Faculty of Veterinary Medicine, Vietnam National University of Agriculture, Hanoi 131000, Vietnam; 4 Department of Veterinary Pharmacology, Joint Faculty of Veterinary Medicine, Kagoshima University, 1-21-24 Korimoto, Kagoshima 890-0065, Japan; k1330977@kadai.jp; 5 Institute of Biotechnology and Food technology, Thai Nguyen University of Agriculture and Forestry, Thai Nguyen 24119, Vietnam; dungnt@tuaf.edu.vn

**Keywords:** *Sophora japonica* bud, antibacterial effect, antioxidant activity, roasting, rutin, quercetin

## Abstract

Roasting is the most common thermal processing method established for *Sophora japonica* (SJ) buds applied as traditional medicines, and it has also been reported to alter several of their therapeutic functions. However, there have been no studies investigating the influences of roasting on the effects of these materials against bacteria. Therefore our study was performed to examine the alterations that this process would induce in SJ buds’ antibacterial properties. Fresh buds were subjected to hot air drying or different roasting methods, as described in Materia Medica, including yellow-, dark yellow-, scorched-, and charred-roasting conditions. Antibacterial effects, total polyphenol and flavonoid contents, antioxidant activities, as well as rutin and quercetin concentrations in methanol extracts obtained from those materials, were then measured and compared. The results showed that dark yellow-roasted SJ buds exerted the strongest antibacterial and antioxidant activities and were also the richest in polyphenol contents. Analysis of rutin and quercetin revealed that, following the increment in heating temperatures up to 240 °C, the reduction in rutin content occurred in a parallel manner to the increment in quercetin content. However, overheating at 300 °C reduced both concentrations. Among the five tested samples, dark yellow-roasted SJ had the highest amounts of quercetin. Furthermore, the comparison of rutin and quercetin in antibacterial effects and antioxidant activities showed that the latter was significantly stronger in both of these functions, suggesting that the increment in quercetin content as a result of heat treatment was responsible, at least in part, for the potentiation of the two therapeutic effects.

## 1. Introduction

*Sophora japonica* (SJ), which is also known as the “Japanese pagoda tree” or “Chinese scholar tree” in English, and “Cây Hoa Hòe” in Vietnamese, is a medicinal and edible plant with a long history of use as both food and traditional medicine in Vietnam [1,2,3]. Among the various plant parts used as herbal medicine, such as flowers, flower buds, fruits, seeds, branches, twigs, leaves, and roots [4], buds are considered the most preferred materials [1,2,3]. Buds also contain the highest concentrations of rutin [5,6], which is considered the main bioactive compound of *Sophora* plants [1,3]. SJ buds are currently applied as an ingredient in various herbal remedies to treat diseases associated with many types of internal hemorrhoids, hemorrhoids, dysentery, epistaxis, bleeding, arteriosclerosis, headache, hypertension, dizziness, inflammation and infections [1,2,3,7]. In addition, they are used in cosmetics [8,9,10], food [9,11], functional foods, and healthy drinks [9,12]. In ethnopharmacology, roasting is generally recommended for the preparation of SJ buds with specific medicinal uses [13,14]. However, the opinion is mainly based on information provided by classical books of Materia Medica, and there is a lack of scientific backgrounds supporting the necessity or advantages of this practice. In addition, it is likely that roasting conditions may vary, and are often established in accordance with each targeted medicinal use [1,2,3,13,14]. Previous research has found that similar conditions of roasting might induce different influences on the pharmacological properties of the same herbal plants, increasing some therapeutic effects, and meanwhile reducing the potencies of others. For example, honey-roasting was observed to significantly enhance the anti-arrhythmic [15] properties of licorice but concurrently decreased its antitussive and detoxifying [16] effects. Interestingly, several studies have shown that roasting under appropriate conditions can significantly enhance the therapeutic properties of herbal materials and induce pharmacological functions that are not observed in raw samples [17,18]. These observations suggest that the influence of roasting on each medicinal effect of a plant material should be investigated separately and in accordance with the targeted therapeutic use. In the case of SJ, modern research has demonstrated that roasting, stir-frying, or parching can induce significant alterations in its pharmacological functions [19,20,21]. For example, while raw SJ is better than processed materials for cooling blood or treating headache and dizziness [20,22], stir-fried or dry-heated materials have been found to alleviate its bitter cold nature [20], enhance its inhibition of xanthine oxidase [21,23], and adenosine deaminase [23], explaining the advantages of applying these processed materials for uric acid-lowering therapies and gout treatment [21]. Furthermore, traditional practitioners believe that roasting with high and prolonged heat to obtain charred materials produces SJ buds with the strongest hemostatic [3,9,20] and antihypertensive [1] effects. Influences induced by roasting on SJ buds have been studied on the changes of phytochemical compounds [21,24], sensory indicators [21,24], and biological values, including polyphenol contents [21], total polysaccharides, and total flavonoids [24], as well as pharmacological functions such as the inhibition of xanthine oxidase enzyme [21]. In contrast, even though the antibacterial properties of SJ have been reported by several researchers [25,26,27,28,29], no studies have investigated the effects of roasting on these properties. Similarly, while alterations in the antioxidant properties of SJ dried under different drying conditions, such as air drying, hot air drying, far-infrared drying, vacuum drying, ultrasound-assisted vacuum drying, and freeze drying, have been evaluated [30,31,32], changes that roasting might induce in this activity remain unexamined. Therefore, our study was performed to investigate the influences that roasting might have on the antibacterial and antioxidant properties of SJ buds, in order to further verify how this traditional method affects their therapeutic potencies. In addition, because alterations in the medicinal effects of plant materials are usually attributed to changes in their bioactive compounds, we also examined the phytochemical constituents of SJ buds processed under different conditions. Rutin and quercetin have been identified as the two main active components responsible for various pharmacological functions of SJ [3,21,33], including antibacterial and antioxidant activities [33]. Previous studies have observed that the most significant changes in the contents of phytochemical compounds that dry thermal processes, such as stir-frying or parching, produced on SJ buds were the alterations in rutin and quercetin constituents [19,21]. In addition, the conversion from rutin to quercetin was found to be responsible for the alterations in several therapeutic effects of roasted or dry-heated SJ materials, such as the inhibition of xanthine oxidase [21,23] or adenosine deaminase [23]. Therefore, we also measured the amounts of these two bioactive compounds, to verify if changes in these constituents are responsible for the differences in antibacterial properties. In addition, other phytochemical and biological values, such as polyphenol contents, total flavonoids, and antioxidant activities, were also examined, as they have been reported to contribute to various pharmacological functions of herbal plants. Taken together, this study aimed to acquire a more scientific understanding of the effects of SJ buds on bacteria and exploit their potential in practice.

## 2. Results and Discussion

### 2.1. Effects of Roasting on Rutin and Quercetin Contents 

**Figure 1:**
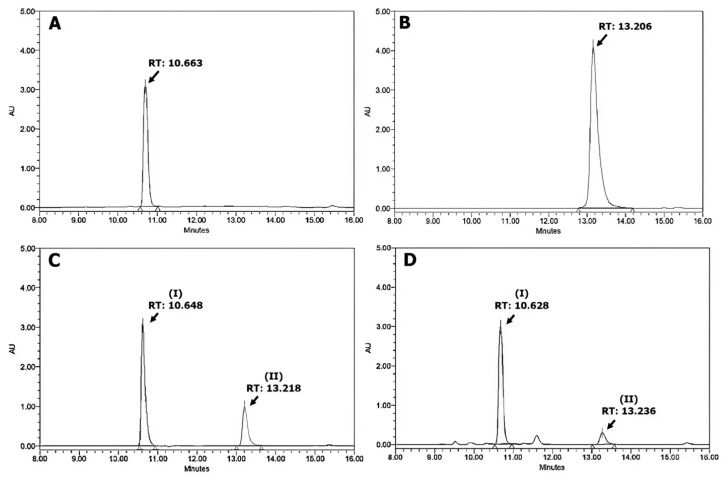
Representative HPLC chromatograms of (**A**) The standard of rutin (4 mg/mL), (**B**) The standard of quercetin (4 mg/mL), (**C**) The mixture standard of rutin (4 mg/mL) and quercetin (1 mg/mL), and (**D**) Methanol extract of non-roasted *Sophora japonica* buds (5 mg/mL; peak I: rutin; peak II: quercetin).

Representative HPLC chromatograms of rutin, quercetin, a mixture of rutin and quercetin, as well as the methanol extract of non-roasted SJ are shown in Figure 1.

The retention time (RT) of rutin was from 10.627 to 10.705 min (Figure 1A), and that of quercetin was from 13.206 to 13.229 min (Figure 1B). Linearity was established by comparing the peak areas with standard reference concentrations. The calibration curves are represented by the equation y=6.275 x−0.7161 (R^2^ = 0.9992) for rutin and y=9.5586 x−0.0676  (R^2^ = 0.9989) for quercetin (where y is the peak response area and x is the concentration). In the chromatography of the mixture of rutin and quercetin, peak I with RT = 10.648 min was identified as rutin, and peak II with RT = 13.218 min was identified as quercetin (Figure 1C). Similarly, for the SJ non-roasted sample, peak I with RT = 10.628 min and peak II with RT = 13.236 min were respectively identified as rutin and quercetin (Figure 1D).

**Figure 2:**
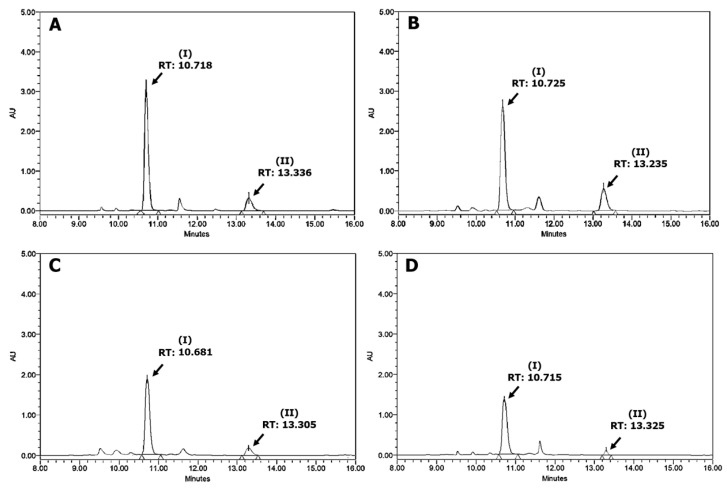
Representative HPLC chromatograms of methanol extracts of (**A**) Yellow-roasted, (**B**) Dark yellow-roasted, (**C**) Scorched-roasted, and (**D**) Charred-roasted *Sophora japonica* buds (5 mg/mL; peak I: rutin; peak II: quercetin).

Representative HPLC chromatograms of yellow-roasted, dark yellow-roasted, scorched-roasted, and charred-roasted SJ buds are shown in Figure 2. In these chromatograms, peaks I with RT ranging from 10.681 to 10.725 min were identified as rutin, while peaks II with RT ranging from 13.305 to 13.336 min were identified as quercetin.

By comparing the areas of peaks in HPLC chromatography profiles of SJ with those of standards, the contents of rutin and quercetin were calculated, based on the weights of the dried extracts (mg/g extract) or initial SJ materials (mg/g dried powder). These are shown in Table 1. 

From Table 1, we observed that there were no significant differences in the yields of crude extracts from oven-dried and roasted samples, which ranged from 32.50% (yellow-roasting) to 32.54% (oven-dried). Our results were in accordance with a previous study that also investigated SJ bud materials collected in Vietnam and observed that the weights of crude extracts ranged from 31.96% to 34.65% of the initial dried powder [34]. When calculated based on dried extracts, the rutin content of oven-dried samples was determined at 784.73 mg/g, equal to 78.47%, similar to the report of Nguyen et al. [35], which determined the content of rutin in dried methanol extracts of Vietnamese SJ buds to range from 70.71 to 80.93%. However, our result was higher than the study of Luong et al. [12], where the amount was determined to range from 69.83 to 71.87%. This difference might be explained by variations in solvents, as they used ethyl acetate and hot water, while our study applied methanol, which has been demonstrated to be preferable for the initial extraction of rutin in SJ buds [35,36]. When calculated based on the weight of the initial materials, the concentrations of rutin and quercetin in non-roasted buds were 255.04 and 14.22 mg/g, respectively, and therefore were equal to 25.50% and 1.42% of dried powders. The results were within the ranges reported by several previous studies, which determined that rutin contents in SJ buds varied from 4.5% [37] to 36.87% [38], and those of quercetin ranged from 0.0295% [39] to 8.99% [40], largely dependent on extracting methods and the origins of the materials [4]. The amount of rutin measured in our study was similar to that of Balbaa et al. [41] and Liu et al. [36], who identified this content at 24.29% or in the range from 12.54 to 29.14%. However, it was lower than the results of Wang et al. [42], who determined this value to range from 29.44% to 37.44%, while, in contrast, it was higher than those reported by Paniwnyk et al. [37], Fan et al. [43], Wang et al. [44], and Li et al. [21], who respectively determined this concentration to be 4.5%, 14.61%, 18.81%, and from 18.3% to 19.1%. Quercetin content in SJ buds observed in our study was similar to that of Fan et al. [43], who reported that the value ranged from 0.5650 to 2.7998%, depending on extracting conditions. However, our result was higher than the research of Li et al. [21], Wang et al. [42], Xie et al. [45], and Xu et al. [46], who respectively identified the concentration to range from 1.04 to 1.15%, from 0.29 to 0.54%, 0.55%, and 0.62%. In contrast, the value was lower than that observed by Wang et al. [47] and Liu et al. [36], who determined this content to be 2.76% and 6.26%, respectively, in their studies. The alterations in rutin and quercetin contents might be partly explained by differences in extracting and measurement methods. In addition, varieties and planting areas could also markedly affect the amounts of active compounds in SJ plants [33].

From Table 1, we observed that roasting at 160 °C did not alter the rutin content. Our results were similar to those of Noda et al. [48], who investigated the effects of roasting on Tartary buckwheat bran and reported that roasting at 160 °C induced no changes in rutin concentrations. These findings were also consistent with several previous studies, which reported that in dry thermal treatments with temperatures less than 200 °C, rutin contents of plant materials were not largely affected [49,50]. However, when heating conditions reached 200 °C, rutin amounts in SJ significantly reduced, and its reduction occurred in a parallel manner to the increment in roasting temperatures. Accompanying the raising of temperatures to 200, 240, and 300 °C, rutin contents in roasted materials correspondingly decreased and were, respectively, equal to only 87.13, 80.48, and 49.35% of the amount measured in non-roasted materials (Table 1). These results were similar to several previous studies, which also reported that the significant decrement in rutin content started when applied temperatures increased to equal or more than 200 °C [48,49,50]. Based on these results, we suggest that 200 °C might be the threshold temperature for the conversion of rutin to quercetin in the dry thermal processing of rutin-containing plant materials, including SJ buds. More specifically, Li et al. [21] also observed a parallelism between the decrements in rutin contents of SJ buds and the increments in temperatures applied for stir-frying conditions. However, the decomposition rate was more severe in their results because rutin loss was more than 90.0% at 280 °C, while in our study, it was 49.35%. The dissimilarities might be explained by the differences in conditions of roasting or stir-frying, as well as the origins and characteristics of SJ bud samples, which could affect the heat stability of the contained active compounds. In contrast to rutin, the amounts of quercetin significantly increased by roasting and reached the maximum content at 200 °C, with a value of 28.26 mg/g dried powder, 1.99-fold higher than the amount contained in non-roasted material (14.22 mg/g). Similar to our results, researchers have reported a parallelism between the decrements in rutin contents and the increments in quercetin contents of SJ buds subjected to dry thermal processes, such as stir-frying [20,21] or parching [19]. More specifically, Li et al. [21] observed that stir-frying under suitable conditions could increase the quercetin concentration of SJ buds to more than 2-fold (from 11.5 mg/g to 25.4 mg/g), which is closely similar to our experiments. However, Ji et al. [20] and Chu et al. [19] reported greater enhancements, where stir-frying or parching respectively increased the amounts of quercetin up to 3.5-fold (from 3.78% to 13.32%) or 4.8-fold (from 10.4 to 50.1 mg/g). The dissimilarities among these investigations might be due to differences in heating methods, as the conditions applied in the stir-frying and parching experiments, including temperature and treatment time parameters, have not been specifically described in previous reports [19,20]. Because the thermal stability of 3-O-glucoside and 3-O-rutinoside is poor [51], high temperatures induce de-glycosylation, which is considered the main reason for the increments in aglycone contents of highly heat-treated plant materials, as they are the thermal degradation products of the glycosides [52]. Besides SJ buds, elevations in quercetin amounts through dry thermal processing, such as simple heating, baking, or roasting, have also been observed to occur simultaneously and in a parallel manner to the decrements of rutin contents in other rutin-rich plant materials, such as Tartary buckwheat flour [53], bran [48], grains [54], blueberry-filled pastries [55], and shells of sweet chestnut [56]. Previous researchers have attributed these enhancements to the de-glycosylation of rutin as a result of heating treatments, which generates quercetin aglycone from rutin and thereby increases its concentration while reducing the amount of its precursor [19,20]. While enzymes are considered the main factors responsible for the cleavage of covalent bonds [57], conditions different from enzymatic activities, such as heating with far-infrared radiation [58] or the Maillard reaction [59], have also been able to cleave the bonds and liberate the compounds. Rutinosidase enzymes are largely deactivated by high temperatures [60,61]. For example, heat treatments above 70 °C have been reported to deactivate these enzymes [60,62], and Noda et al. [48] also observed that rutinosidase became denatured when plant materials were subjected to 200 °C heating. In addition, dissimilar to hydrothermal methods, there was no addition of water, which is essential for the activations of rutinosidase enzymes, in the process of dry thermal methods employed in our study and previous research, as SJ was subjected to roasting, stir-frying, and parching [19,20,21]. Therefore, it is likely that factors different from enzymatic activities, such as high-temperature-induced degradation or the Maillard reaction, are mainly responsible for the enhancements in quercetin contents observed in our study and previous research on SJ buds [19,20,21]. However, at temperatures of 240 °C or higher, both rutin and quercetin amounts decreased simultaneously. Ghimeray et al. [63] also observed that there was a temperature threshold for each material sample, that in which, quercetin contents were enhanced in a parallel manner to the increment of temperatures, and overheating to more than that threshold level would decrease quercetin concentrations. In our study, the decomposition rate was most drastic at 300 °C, when the contents of rutin and quercetin were respectively reduced to 125.85 mg/g and 6.88 mg/g, equal to only 49.35% and 48.38% of the amounts in non-roasted materials. The sharp decrements in concentrations of both rutin and quercetin when samples were subjected to very high temperatures in dry heating treatments has also been observed with other rutin-rich materials [48,53]. Buchner et al. [64] investigated the thermal-induced degradation in the quercetin and rutin model system, and reported that the former showed faster degradation than the latter when they were subjected to the same heating conditions. Therefore, Fujita et al. [61] explained that when heating temperatures reached the threshold at which the degradation of quercetin itself becomes smaller than the equivalent decrease in rutin, both contents would be reduced. Similar to our study, previous research also reported that while rutin always remarkably decreased with prolonged dry heating times, such as with baking or stir-frying conditions, significant increments in quercetin concentrations were only observed under appropriate conditions [21,53]. In an investigation with SJ buds subjected to stir-frying, researchers also reported that, over high heat and prolonged times, both rutin and quercetin amounts in the materials were reduced [21]. However, the decomposition process was more severe in their study, as charred-roasting could destroy 98.5% of rutin (2.75 mg/g vs. 183 mg/g) and 73.8% (6.40 vs. 24.4 mg/g) of quercetin in SJ buds [21]. Tong [65] also reported the drastic reduction of rutin content, stating that 77.7% of the amount contained in SJ buds was destroyed when roasting the materials to char. However, Do et al. [2] and Ji et al. [20] observed less severe rates of decomposition, with rutin contents in charred-SJ buds reduced by 46.69% and 51.89%, respectively, resembling our observation (50.65%). Besides rutin, the quercetin amount also remarkably decreased under overheated conditions, as its concentration in charred-SJ was only 6.88 mg/g, reduced by 75.7% compared with the highest value obtained under dark yellow-roasting conditions (28.26 mg/g). Our result was in accordance with the study of Li et al. [21], which observed that stir-frying to char produced SJ buds with a small amount of quercetin: 6.40 mg/g, meaning that the treatment had destroyed 74.8% of the quercetin contained in SJ buds when compared with the highest value obtained under optimal conditions (25.4 mg/g). In contrast, Ji et al. [20] observed the opposite outcome with stir-frying to char, reporting that this treatment yielded the highest amount of quercetin (13.2%), which was 3.49- and 1.98-fold higher than the amounts contained in non-fried (3.78%) and dark yellow-fried (6.65%) SJ buds. The dissimilarities among investigations might be mainly due to the heating conditions, as most of the studies followed the descriptions in classical books of Materia Medica to process SJ buds and separate them into different types, such as dark yellow stir-frying or charred-frying treatments [20,21]. However, traditional knowledges mainly relies only on the changes in sample appearance, including color and flavor, to process the materials; therefore, treatment parameters, such as temperatures and heating time, varied among investigations [19,20,21]. In addition, the origins and characteristics of the examined SJ materials were also dissimilar. Previous studies have reported that the influences of roasting were varied and highly dependent on the nature of the plant samples [48], as different influences were observed even when similar treatments were applied to materials from the same trees. For example, while roasting at 160 °C did not alter rutin contents in buckwheat flour and bran, it significantly reduced these amounts to only one-sixth of the raw materials when the same conditions were applied to buckwheat groats [66]. Similarly, the parallelism between increments in quercetin and decrements in rutin contents was only observed when applying roasting treatment to the inner and outer shells of chestnut but did not occur when applied to the seeds of chestnut [56]. Considering the results altogether, we suggest that dark yellow-roasting was the method that yielded the highest content of quercetin for SJ buds because less heating, such as in the case of yellow-roasting, did not generate the conversion of rutin to quercetin, while further heating to scorched or charred materials led to the decomposition of active compounds, thereby reducing both rutin and quercetin amounts.

### 2.2. Effects of Roasting on Antibacterial Effects of SJ Buds

#### 2.2.1. Effects of Roasting on MIC Values

MIC values of SJ methanol extracts and purified compounds against three gram-positive (gram (+)) bacteria, including *Bacillus cereus* (*B. cereus*) ATCC 11778, *Bacillus subtilis* (*B. subtilis*) ATCC 6633, and *Staphylococcus aureus* (*S. aureus*) ATCC 25923, and three gram-negative (gram (−)) bacteria, including *Escherichia coli* (*E. coli*) ATCC 25922, *Pseudomonas aeruginosa* (*P. aeruginosa*) ATCC 9027, and *Salmonella enterica* subsp. *enterica* serovar Typhimurium (*S*. *typhimurium*) ATCC 13311, are shown in Table 2. 

From Table 2, we observed that non-roasted SJ buds exerted effects on all examined gram (+) and gram (−) bacteria. The results were in accordance with previous research, which also reported the broad-spectrum inhibitory effects of SJ flower and bud materials on both gram (+) and gram (−) bacteria [25,27,67]. MIC values in our study ranged from 15.6 to 4000 µg/mL, in accordance with those obtained in previous research [25], which reported that the MICs ranged from 0.125 to 4 mg/mL, or from 125 to 4000 µg/mL. In comparison with other SJ materials, the MICs of methanol extracts determined for SJ buds in our study were lower than those for methanol extracts obtained from SJ fruits and stems, as their MICs against both gram (+) and gram (−) bacteria were in the range from 0.7 to 11.25 mg/mL, or from 700 to 11250 µg/mL [68]. These observations indicate that buds are more potent than other SJ plant parts in antibacterial activities. In addition, we observed that gram (+) bacteria were more sensitive than gram (−) bacteria to both SJ extracts and isolated compounds, as shown by the lower MIC values (Table 2). These results were in accordance with several previous studies, which also reported the less sensitive gram (−) bacteria with SJ materials [69] and quercetin [70]. In general, lower MIC values against gram (+) bacteria are often reported for plant extracts and compounds compared to gram (−) bacteria. This has been attributed to the thick outer murein layer in the structure of gram (−) bacteria, which prevents the entry of inhibitors [71]. From Table 2, we observed that *S. aureus* was the most sensitive, as evidenced by the lowest MIC values for all tested extracts and compounds. This was similar to a previous study, which also reported that, among all tested bacteria, *S. aureus* species had the highest sensitivity to SJ materials [29,69] and quercetin [70]. In the case of gram (−) bacteria, *E. coli* ATCC 25922 was the most sensitive bacterium to both SJ extracts and quercetin, as shown by the lower MICs. This resembled the studies of Yao et al. [29] and Nitiema et al. [72], which respectively reported the high sensitivity of *E. coli* species to SJ materials and quercetin.

When investigating isolated compounds, we found that both rutin and quercetin showed inhibition of bacteria. Researchers have reported that the antibacterial properties of rutin were mediated via inhibition of efflux pumps and interference with biofilm formation [73]. On the other hand, quercetin’s effects on bacteria have been attributed to alterations in cell permeability, disruption of cell walls, inhibition of nucleic acid synthesis, and the reduction of enzyme activity [74]. However, quercetin exerted better inhibition than rutin, as, regardless of the examined bacterial strains, the former always had lower MIC values. In accordance with our results, Rauha et al. [75] reported that quercetin showed effects against both gram (+) and gram (−) bacteria, and Arima et al. [76] observed that rutin, at concentrations up to 1000 µg/mL, exerted no inhibition on both *B. cereus* and *Salmonella enteritidis*. In addition, previous studies have also found that quercetin was more potent than rutin [75,77]. With the consideration of each bacterial strain, the MIC of rutin against *S. aureus* ATCC 25923 was 2000 µg/mL, comparable to but slightly higher than that of a previous study [78], which determined this MIC at 1677.26 µM, equal to 1024 µg/mL. Similarly, the MIC of quercetin against *S. aureus* ATCC 25923 was 250 µg/mL, resembling but slightly lower than the result obtained by Yan et al. [78] and Jaisinghani [79], who reported this value at 1323.45 µM, which was equal to 400 µg/mL. From Table 2, we observed that quercetin exerted better effects on gram (+) than gram (−) bacteria, as evidenced by the lower MICs (ranging from 250 to 500 µg/mL for gram (+) vs. from 1000 to 2000 µg/mL for gram (−) strains). Similarly, Rauha et al. [75] reported that quercetin was more potent against gram (+) bacteria.

When comparing MICs obtained with different SJ bud samples on each individual bacterium, we observed that yellow-roasting did not alter the effects, as the MICs of the materials were the same as those of non-roasted SJ against all bacteria. In contrast, it is noticeable that inhibitions were significantly enhanced with dark yellow-roasted and scorched-roasted materials because, regardless of the examined bacterial strains, extracts from these two materials always exerted the strongest inhibition, as evidenced by the lowest MIC values among all five samples. However, in the comparison between the two materials, dark yellow-roasted ones were superior, as the MICs were lower than those of scorched-materials in cases of activities against three gram (+) bacteria and the *E. coli* ATCC 25922 strain. These results suggested that the two processing methods effectively increased the antibacterial properties of SJ. In addition, the enhancements were mediated, at least in part, via the conversion of rutin to quercetin, as Table 2 showed that the latter was significantly stronger than the former in inhibitions against all tested bacteria (MICs ranged from 250 to 2000 µg/mL vs. MICs ranged from 2000 to 4000 µg/mL). Furthermore, dark yellow-roasted samples, which had the highest amount of quercetin, also exerted the strongest effects. In contrast, roasting to char significantly reduced the potencies of SJ, as MICs were increased to equal or even higher than those of non-roasted materials with all bacteria. The reduced effectiveness might be partly explained via the decrements in both quercetin and rutin contents, which had been observed to exert inhibition against all examined bacteria (Table 2), and therefore, the decline in their amounts would lead to the reduction of extracts’ total effects.

#### 2.2.2. Effects of Roasting on Inhibitory Zones

**Figure 3:**
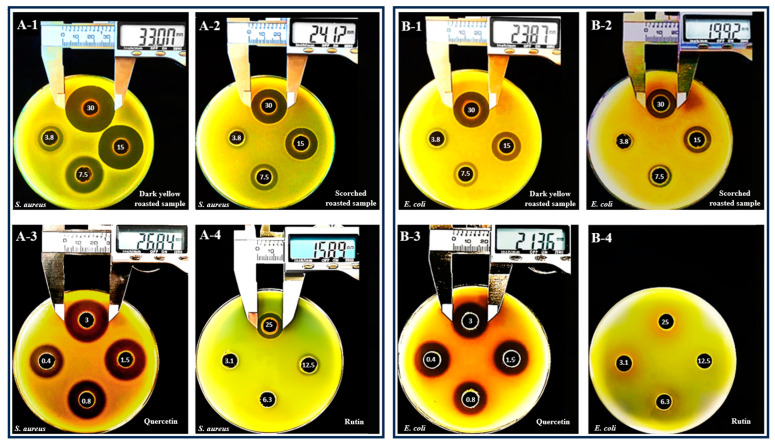
Inhibitory zones induced by methanol extracts of *Sophora japonica* buds and isolated quercetin and rutin on (**A**) *Staphylococcus aureus* ATCC 25923 and (**B**) *Escherichia coli* ATCC 25922. (**A-1**) methanol extract of dark yellow-roasted, (**A-2**) methanol extract of scorched-roasted, (**A-3**) quercetin, (**A-4**) rutin on *Staphylococcus aureus* ATCC 25923; and (**B-1**) methanol extract of dark yellow-roasted, (**B-2**) methanol extract of scorched-roasted, (**B-3**) quercetin, (**B-4**) rutin on *Escherichia coli* ATCC 25922. Concentrations of an extract applied in four tested wells of each petri dish were 30 mg/mL, 15 mg/mL, 7.5 mg/mL, and 3.8 mg/mL; concentrations of quercetin applied in four tested wells of each petri dish were 3 mg/mL, 1.5 mg/mL, 0.8 mg/mL, and 0.4 mg/mL; while those of rutin were 25 mg/mL, 12.5 mg/mL, 6.3 mg/mL, and 3.1 mg/mL.

Inhibitory zones (mm) induced by methanol extracts of SJ buds and isolated compounds, including quercetin and rutin, are shown in Table 3 and Figure 3.

From Table 3, we observed that SJ extracts were able to induce effects on both gram (+) and gram (−) bacteria but exerted stronger activities on gram (+) ones, as shown by the significantly larger inhibitory zones. In addition, while gram (+) strains were sensitive to all SJ extracts except for the case of charred materials, gram (−) ones were only inhibited by those from dark yellow- and scorched-SJ buds. These results resembled those obtained in broth dilution methods and therefore confirmed the higher susceptibility of gram (+) bacteria. In addition, this was in accordance with the previous study of Yao et al. [29], which also applied diffusion methods to investigate and reported that SJ extracts were able to induce inhibitory zones on both gram (+) and gram (−) bacteria, with diameters ranging from 3.00 to 15.67 mm. Similar to the results of the broth dilution methods, we observed that *S. aureus* ATCC 25923 was the most sensitive bacterium, as regardless of the tested SJ bud materials and except only for the case of charred samples, this strain always had the largest inhibitory zones, with diameters induced by extracts at the highest concentration (30 mg/mL) ranging from 9.04 ± 0.05 to 22.1 ± 1.25 mm (Table 3 and Figure 3(A-1,A-2)). Our results were similar to those of Yao et al. [29] and Si et al. [69], as they also found the highest susceptibility of *S. aureus* species against SJ, with zones of 15.67 mm and 18.20 mm. Among the examined gram (−) bacteria, *E. coli* ATCC 25922 was the most sensitive, as dark yellow and scorched materials induced the largest zones on this strain, with diameters of the highest extract concentrations (30 mg/mL) being 12.63 ± 1.08 and 9.53 ± 0.40 mm, respectively, for the two materials (Table 3 and Figure 3(B-1,B-2)). The zones against *S*. typhimurium were 8.10 ± 0.22 and 4.70 ± 0.34 mm, while those against *P. aeruginosa* were 9.04 ± 0.23 and 5.10 ± 0.23 mm (Table 3). Yao et al. [29] also observed similar results, reporting that *E. coli* was the most sensitive gram (−) species to SJ, as the strain had a significantly larger zone (15.50 mm) than those of *S.* typhimurium (6.17 mm) and *P. aeruginosa* (3.17 mm). 

**Figure 4:**
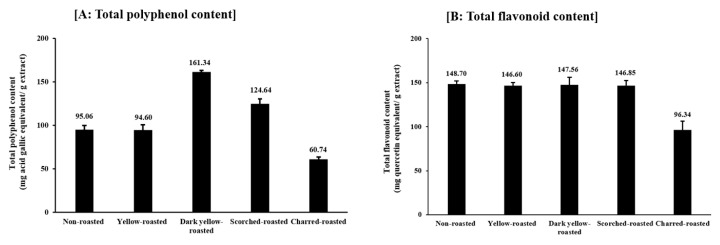
Results on total (**A**) polyphenol and (**B**) flavonoid contents of methanol extracts from non-roasted and roasted *Sophora japonica* buds. Results are expressed as means ± standard deviation (SD) of three tests.

When investigating isolated compounds, we observed that quercetin exerted better inhibition than rutin. This was similar to several previous studies, which also applied diffusion methods to examine and report the superior effects of quercetin against bacteria [75,76,77]. Even though both compounds were effective against gram (+) bacteria, quercetin was significantly stronger, as evidenced by the larger inhibitory zones against all tested gram (+) bacteria, including *B. cereus*, *B. subtilis*, and *S. aureus* (Table 3 and Figure 3(A-3) vs. Figure 3(A-4)). In addition, quercetin had broader-spectrum antibacterial properties than rutin, as the latter induced inhibitory zones only on gram (+) bacteria, while the former could also inhibit gram (−) bacteria, including *E. coli* ATCC 25922 (Figure 3(B-3) vs. Figure 3(B-4)), *S*. typhimurium ATCC 13311, and *P. aeruginosa* ATCC 9027. *S. aureus* was found to be the most sensitive species against quercetin, induced zones with the largest diameters (15.68 ± 1.02 mm at 3 mg/mL, Figure 3(A-3)), resembling the results obtained with broth dilution methods. Similar to our study, Latos-Brozio et al. [77] also observed the highest sensitivity to quercetin in *S. aureus* species, with inhibitory zones ranging from 25 to 28 mm. Among the examined gram (−) bacteria, *E. coli* was the most sensitive to quercetin, as evidenced by the largest inhibitory zone (10.43 ± 0.82 mm at the highest examined concentration, 3 mg/mL, Figure 3B-3), while those obtained for the cases of *S. typhimurium* and *P. aeruginosa* were only 8.75 ± 0.43 and 7.75 ± 0.35 mm. This resembled the results of Nitiema et al. [72], who also reported that *E. coli* was more sensitive to quercetin than *Salmonella* (zone diameters of 8.50 ± 2.12 mm vs. no inhibition).

When comparing the effects of extracts from different SJ samples on each individual bacterium, we observed that roasting significantly altered the inhibitory effects, with a trend similar to those observed in broth dilution methods. Firstly, the results showed that regardless of the examined bacterial strains, dark yellow- and scorched-roasted materials always exerted the strongest effects, as shown by the significantly larger inhibitory zones (Table 3 and Figure 3(A-1,A-2,B-1,B-2)). In addition, only these two materials exerted effects on gram (−) bacteria (Table 3 and Figure 3(B-1,B-2)), while the remaining samples were not able to induce inhibition on them. These observations resembled the results from the broth dilution methods, confirming that roasting materials to dark yellow or scorched colors could effectively increase SJ’s antibacterial properties. However, the effects of dark yellow-roasted samples were superior, as they induced larger inhibitory zones than scorched materials for both gram (+) (Table 3 and Figure 3(A-1) vs. Figure 3(A-2)) and gram (−) bacteria (Table 3 and Figure 3(B-1) vs. Figure 3(B-2)). In contrast, roasting to char reduced the potencies, as materials became ineffective against all bacteria. Interestingly, we also found that changes in the inhibitory effects of SJ occurred in a parallel manner to the alterations in rutin and quercetin constituents. When normalizing the concentrations of extracts applied in each well of agar diffusion methods to the contents of quercetin, the range of tested doses from 30 to 3.75 mg extract/mL were converted from 1.31 to 0.16 mg quercetin/mL for non-roasted; 1.33 to 0.17 mg quercetin/mL for yellow-roasted; 2.61 to 0.33 mg quercetin/mL for dark yellow-roasted; 1.76 to 0.22 mg quercetin/mL for scorched-roasted; and 0.64 to 0.08 mg quercetin/mL for charred-roasted materials. Similarly, the normalized results for rutin were from 23.54 to 2.94 mg rutin/mL; 23.46 to 2.93 g rutin/mL; 20.51 to 2.56 mg rutin/mL; 18.95 to 2.37 mg rutin/mL; and 11.62 to 1.45 mg rutin/mL for the five SJ materials. Because the tests with isolated quercetin and rutin on the same set of bacteria applied to SJ extracts revealed that at these doses, quercetin was able to exert clear inhibitory zones on all bacteria (Table 3, Figure 3(A-3,B-3)), and rutin also significantly inhibited the gram (+) ones (Table 3, Figure 3(A-4)), we concluded that the containment of these two compounds contributed to the total effects of the extracts. In addition, the conversion of rutin to quercetin also played a role in the enhancement of antibacterial properties in dark yellow- and scorched-roasted materials, because quercetin was significantly stronger than rutin in this potency. Specifically, quercetin was able to exert effects on gram (−) bacteria, while rutin induced no inhibition on these bacteria (Table 3 and Figure 3(B-3) vs. Figure 3(B-4)). Furthermore, quercetin’s effects against gram (+) bacteria were also remarkably stronger, as evidenced by the larger inhibitory zones (Table 3 and Figure 3(A-3) vs. Figure 3(A-4)). Therefore, it is likely that the highest amounts of quercetin contained in dark yellow SJ partly contributed to the samples’ strongest antibacterial effects. In addition, thermal decomposition of both rutin and quercetin as a result of overheating to char was responsible, at least in part, for the materials’ reduced effectiveness. With charred-SJ buds, the normalized contents of quercetin and rutin contained in wells employed in diffusion methods reduced to only 0.64 to 0.08 mg/mL for quercetin, and 11.62 to 1.45 mg/mL for rutin; as shown in Table 3, these two compounds were not able to induce inhibition against any bacteria when being applied at these concentrations. These results indicated that the drastic decline in the amounts of rutin and quercetin, down to inactive levels, was partly responsible for charred-SJ buds’ ineffectiveness against bacteria.

Interestingly, we observed that in both the broth dilution and agar well diffusion methods, and regardless of the examined bacteria, crude methanol extracts from dark yellow-roasted and scorched-roasted SJ were always able to induce stronger effects than isolated rutin or quercetin, as evidenced by lower MIC values (Table 2) and larger inhibitory zones (Table 3). Our results were similar to several previous studies, which also demonstrated that whole extracts could exert better antibacterial potencies than isolated bioactive ingredients, suggesting the important roles of other minor compounds in these plants’ total effects [80,81]. Synergisms among different constituents have been proposed for various therapeutic functions, thus supporting the concept that employing whole plant materials is more advantageous than applying only isolated active ingredients, which underpins the philosophy of using herbal medicine in practice [82]. Previous studies also demonstrated that while higher contents of major active constituents are generally considered to represent higher therapeutic qualities of plant materials, they might not be able to exert equal effects as whole extracts if applied as isolated agents [80]. In the case of SJ, even though rutin and quercetin have been considered the most noticeable antibacterial compounds [33], researchers have also observed that other flavonoids, like genistein and kaempferol, or other phenolic group in *Sophora* trees, such as dihydroxyflavones, were also significantly potent in their effects against bacteria [83]. Future research is therefore necessary to investigate the roles of other compounds, as well as the changes in their constituents induced by roasting, to further elucidate the mechanism responsible for the antibacterial properties of SJ buds and widen their applications in bacterial infections.

Considering the results obtained with both the broth dilution and agar diffusion methods, it is confirmed that treatments of roasting to dark yellow or scorched colors enhanced the antibacterial properties of SJ buds, with the dark yellow samples being superior and thus considered the best material against bacteria. On the other hand, yellow-roasting did not alter the potency of SJ buds, while over-roasting to char, in contrast, significantly reduced the effects. In addition, changes in the antibacterial properties of SJ might be attributed, at least in part, to alterations in the rutin and quercetin contents as the result of heating at different conditions.

### 2.3. Effects of Roasting on Total Polyphenols, Total Flavonoids, and Antioxidant Activities of SJ Buds

Results on total polyphenols and flavonoid contents of non-roasted and roasted SJ bud materials are shown in Figure 4.

When calculated based on the weights of dried extract, phenolic and flavonoid contents measured in non-roasted SJ buds were 95.06 GAE mg/g (Figure 4A) and 148.70 QE mg/g (Figure 4B), and therefore were in accordance with those of Zhang et al. [84], who reported these values at 99.31 GAE mg/g and 151.86 mg QE/g for SJ alcohol extracts. However, our results were higher than those of Yang et al. [85], who quantified these values at 6.02 GAE mg/g and 1.66 mg QE/g extract, while, in contrast, they were lower than those reported by Wang et al. [86], who determined the phenolic content at 255 GAE mg/g extract. The discrepancies might be due to differences in the origins of tested materials, applied solvents, and investigated methods, as phytochemical values largely depend on these factors [84]. The polyphenol content of non-roasted SJ buds in our study was 95.06 GAE mg/g extract, higher than those detected in the stems and leaves of *Sophora* trees, which were respectively determined at 22.09 and 18.45 mg/100 g extract [87]. In contrast, the value was found to be lower than those measured in other plant parts of the same SJ tree, including barks, fruits, and seeds, as Saffidine et al. [68] reported the maximum total phenolic contents of these materials at 316.19 GAE mg/g, 271.71 GAE mg/g, and 310.16 GAE mg/g respectively. Nevertheless, the flavonoid content of SJ buds observed in our study (148.70 QE mg/g, Figure 4B) was always higher than the values obtained in all other plant parts, such as barks, fruits, seeds [68], stems and leaves [87], confirming that buds are the materials richest in flavonoid compounds in SJ trees [41,88].

From Figure 4A, we observed that while yellow-roasting did not alter the total polyphenol contents, dark yellow- and scorched-roasting significantly increased the values. The enhancement was the most remarkable in the case of dark yellow-roasted materials, in which the phenol concentration was elevated to 161.34 GAE mg/g, increasing up to 69.7% compared with the non-roasted SJ buds (95.06 GAE mg/g). However, roasting to char reduced the content to 60.74 GAE mg/g, which was equal to only 63.9% of the non-roasted samples (95.06 GAE mg/g). In opposite to phenolic contents, roasting did not alter the total flavonoid values, except in the case of extreme heat in charred materials (Figure 4B). Similar to our study, previous researchers also observed that heating induced more substantial decrements in total phenolics than flavonoids in some plant materials [89]. These results might be explained via the structures of plant flavonoids, which are usually linked by glycosidic bonds through sugar ligands, such as glucose, rhamnose, or galactose. Therefore, in general they are more stable during heat treatment compared with other phenolic compound groups [89]. Our results indicated that roasting SJ buds up to the scorched level induced fluctuations in the amount of each flavonoid, including alterations in rutin and quercetin concentrations, but retained the overall flavonoid content. Sharma et al. [90] also observed the same phenomenon in onions subjected to dry thermal processes, reporting that while the amount of each flavonoid varied with heating, the total flavonoid contents of materials remained unchanged.

Results on antioxidant activities of SJ bud materials, standard references, and isolated compounds, represented by IC_50_ values and assessed by DPPH and ABTS assays, are shown in Table 4.

From Table 4, we observed that the IC_50_ of standard reference Trolox in the DPPH and ABTS assays was determined to be 3.75 ± 0.03 µg/mL and 2.91 ± 0.04 µg/mL, respectively, resembling the previous study [91], which identified these values as 3.765 ± 0.083 µg/mL and 2.926 ± 0.029 µg/mL. Similarly, the IC_50_ values of gallic acid in DPPH and ABTS assays were 2.75 ± 0.06 µg/mL and 1.97 ± 0.02 µg/mL, which were similar to the studies of Scherer et al. [92] and Brighente et al. [93], as they reported the values in DPPH as 2.80 µg/mL and 2.60 µg/mL. The IC_50_ of the methanol extract from non-roasted SJ buds was 14.91 ± 0.33 µg/mL and 8.22 ± 0.11 µg/mL in the DPPH and ABTS assays, in accordance with the study of Wang et al. [86], who reported the DPPH-IC_50_ of the SJ alcohol extract as 14.46 µg/mL. In cases of isolated compounds, including quercetin in rutin, their IC_50_ values in the DPPH and ABTS assays were 2.93 ± 0.32 and 9.65 ± 0.06 µg/mL, and 2.04 ± 0.02 and 4.54 ± 0.02 µg/mL, respectively, resembling with a slight difference to the report of Lee et al. [94]. With SJ methanol extracts, our results revealed that while yellow-roasting did not alter the antioxidant potencies, dark yellow- and scorched-roasting remarkably increased the activities, as evidenced by the significant decrement in IC_50_. The elevation was most remarkable with dark yellow-roasted materials, as the IC_50_ was decreased to the lowest values in both the DPPH and ABTS assays (9.31 ± 0.10 µg/mL and 5.25 ± 0.05 µg/mL, respectively). However, roasting SJ buds to char reduced the activities, as both the DPPH- and ABTS-IC_50_ values increased to even higher than those of the non-roasted materials (20.80 ± 0.26 vs. 14.91 ± 0.33 in DPPH and 9.70 ± 0.14 µg/mL vs. 8.22 ± 0.11 µg/mL in ABTS assays). In addition, we observed that the alteration tendency induced by roasting in the antioxidant activities of SJ was similar to that in polyphenol contents. Specifically, roasting to dark yellow and scorch enhanced the antioxidant effects because the IC_50_ values were respectively decreased to only 62.4% and 85.6% in DPPH, as well as 63.9% and 81.0% in ABTS assays, compared to those of the non-roasted materials. This was therefore correlated with the elevation of phenolic contents, as those in the dark yellow- and scorched-roasted samples were also observed to increase, reaching to 169.7% and 131.1% higher than the amounts contained in non-treated materials. Furthermore, roasting to char reduced both the antioxidant and polyphenolic values, as this extreme heating process increased the IC_50_ values to even higher than those of the non-roasted materials in both DPPH and ABTS assays, while also decreasing the polyphenol content to only 63.9% of the amount contained in non-roasted samples. Phenolic compounds are mainly responsible for the antioxidant activity in most plant materials [95]. Therefore, the similarity in changing tendencies of total phenolic compounds and antioxidant potencies indicated that the alterations in phenol contents as a result of heat treatment of SJ buds were the major contributors to the changes in their antioxidant capacities. In addition, even though total flavonoid contents remained unchanged until the materials were roasted to scorch, alterations in the compositions of flavonoid compounds, such as the conversion of rutin to quercetin, also played a role in the enhancement of SJ antioxidant capacities. From Table 4, we observed that quercetin, the content of which was noticeably enhanced by appropriate roasting, was also markedly more potent in antioxidant activities than its precursor rutin. Specifically, the DPPH- and ABTS-IC_50_ values of rutin were 3.3- and 2.2-fold higher than those of quercetin (9.65 ± 0.06 vs. 2.93 ± 0.02 µg/mL and 4.54 ± 0.02 vs. 2.04 ± 0.02 µg/mL). These results were in accordance with reports by Lee et al. [94], Aliaga et al. [96], and Rosiak et al. [97], and therefore further confirmed that quercetin was stronger as an antioxidant. Previous research has also observed that the biological activities of flavonoids are structure-dependent; for example, aglycones are more effective antioxidants than their corresponding glycosides [98], and flavonoids with free 3-OH groups, such as quercetin, are excellent antioxidants [99]. The presence of disaccharide in rutin changes the properties of this compound in relation to quercetin [100] and significantly affects the antioxidant activities in DPPH and ABTS tests [101]. In addition, heating might also improve the antioxidant capability of plant materials by increasing the release of phenolic constituents [102] and by the formation of novel compounds, such as Maillard reaction products with antioxidant activities [103]. However, when roasting SJ buds to char, both polyphenol and antioxidant properties were decreased drastically. A similar phenomenon has been observed with other plant materials, such as onions [90] and coffee [95], where the values were also enhanced under appropriate heating conditions, while prolonged treatment and extremely high temperatures reduced both of them. For the case of SJ buds in our study, the decrement in phenolic contents and antioxidant activities of charred materials might be partly attributed to the thermal decomposition of quercetin and rutin, as they are both polyphenol compounds and antioxidants, and their amounts were also intensely reduced by roasting to char treatment.

Overall, our study has demonstrated that roasting under appropriate conditions could significantly enhance the antibacterial and antioxidant properties of SJ buds. In addition, the enhancements are mediated, at least in part, via the conversion of rutin to quercetin, as the latter is significantly more potent in both activities. Even though rutin is considered the main bioactive constituent in SJ buds, and Pharmacopeias [3,104] have established this compound as the required chemotype, as well as applying its content as the standard to evaluate the quality of SJ materials, researchers have demonstrated that quercetin is also important in various therapeutic functions of SJ [4]. Furthermore, quercetin has been observed to exert higher activity than rutin in several pharmacological effects, such as the inhibition of α-amylase and α-glucosidase [105], xanthine oxidase [21], and adenosine deaminase [21,23], suggesting that this compound plays an essential role in the treatment effects of SJ for diabetes, postprandial hyperglycemia, and gout. The conversion of rutin to quercetin has also been reported to enhance therapeutic and biological activities, such as antioxidant, anti-inflammatory, and anti-adipogenic effects [106]. In our study, roasting to dark yellow color is determined to be the optimal condition to potentiate antibacterial and antioxidant activities, as well as to enhance the total polyphenol content and the amount of quercetin. These results suggest that the method should be employed in the preparation of SJ buds aimed to be applied for antibacterial effects, as well as other therapies associated with antioxidant or quercetin functions. Moreover, further research is necessary to standardize roasting conditions, such as specifying important parameters like temperature and heating time, to ensure the beneficial effects obtained through this treatment. It is likely that traditional knowledge of Materia Medica is mainly based on changes in sample appearance to establish and classify roasting conditions, which makes the induced influences and the therapeutic properties of roasted materials inconstant. Previous researchers have also suggested that roasting is able to produce novel compounds that are different from those in raw materials, indicating that separate establishments of quality control for roasted materials are necessary because the therapeutic functions, as well as bioactive compounds, might differ from those of raw materials [18].

## 3. Materials and Methods

### 3.1. Plant Materials and Extraction

**Figure 5:**
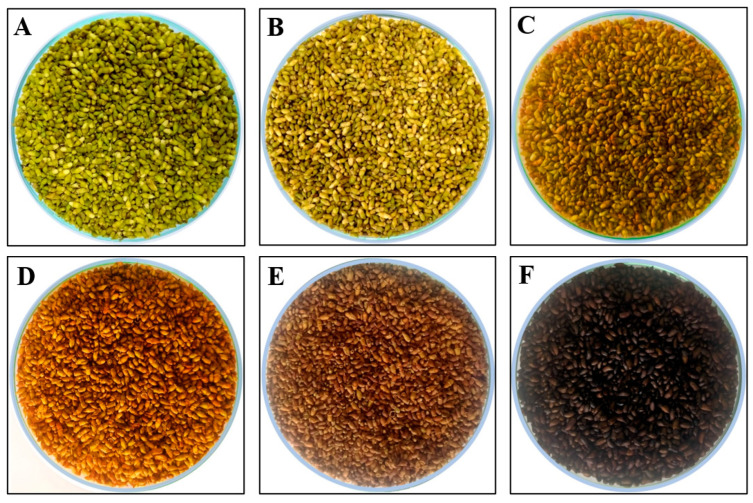
Photographs of typical fresh, oven-dried, and roasted *Sophora japonica* buds. (**A**) Fresh, (**B**) Non-roasted (oven-dried), (**C**) Yellow-roasted, (**D**) Dark yellow-roasted, (**E**) Scorched-roasted, and (**F**) Charred-roasted bud materials.

SJ buds were supplied by Vuon Duoc Lieu Herbarium, Vietnam National University of Agriculture (Hanoi, Vietnam). The plant identity was confirmed by Dr. Tho Thi Bui, based on the voucher specimen that has been deposited at the Vietnam National University of Agriculture. The provider certified the products met the Vietnam National Standards for herbal materials used as medicine, established in the Vietnamese Pharmacopoeia by the Vietnam Ministry of Health, which requires that the content of rutin in dried materials is not less than 20% [3]. Bud materials were either dried by a hot air oven at 40 °C for 2 d or by roasting. The most ideal drying temperature for glycosides was 45–50 °C [107]; however, 40 °C was selected in our set of experiments because hot air drying at this temperature has been reported to not activate hydrolysis enzymes in SJ buds, and therefore rutin was not converted to quercetin during the drying process at this temperature [108]. The methods of roasting were performed following the “General Guidance on the Traditional Processing of medicinal plants” (Vietnam Ministry of Health, Circular Number 30/2017/TT-BYT, issued 2017) [109], with some modifications. Characteristics established for each type of roasting were followed according to the descriptions in Vietnamese traditional books of Materia Medica [2,13,14], including: (1) yellow-roasting: the color changes to bright yellow, and the smell changes from fresh to a mild roasted fragrance; (2) dark yellow-roasting: the color turns dark yellow with a strong roasted fragrance; (3) scorched-roasting: the color turns brown with a slightly burning smell; and (4) charred-roasting: the color turns black with a clear burning smell. To obtain the characteristics described for these types of roasting, our experiments employed a herbal roasting machine (Model: TMND-B06, Tan Minh instruments, Hanoi, Vietnam), and buds were processed at temperatures of 160 °C, 200 °C, 240 °C, and 300 °C, to respectively obtain yellow, dark yellow, scorched, and charred SJ materials. The speed of drum rotation was 30 rpm, and the heating time was 30 min in all treatments. The water content of materials dried by hot air oven and roasted under the conditions of yellow, dark yellow, scorched, and charred samples were 10.5%, 10.8%, 10.6%, and 9.8%, respectively. This indicated that all five drying methods were able to produce materials with moisture less than 11%, and therefore reached the criterion about levels of water contents established for dried SJ buds in Pharmacopeias [3,104]. Typical SJ fresh buds and samples subjected to drying with a hot air oven or different conditions of roasting are shown in Figure 5.

Non-roasted and roasted materials were then ground in to powder before passing through a 100-mesh screen. Extracting process was performed following the optimal method established for the extraction of main bioactive compounds from SJ buds, as reported by Liu et al. [36]. Specifically, extraction conditions were as follows: extraction solvent: 100% methanol; particle size: 100 mesh; extraction frequency: 1; liquid-to-solid ratio: 50:1; microwave power: 287 W; and extraction time: 80, applying the MARS 6 microwave reaction system (CEM Co., Ltd., Matthews, NC, USA). The mixtures were then centrifuged at 10,000× *g* for 30 min and filtered through grade No. 2 qualitative paper (Advantec MFS Inc., Dublin, CA, USA) to remove all precipitates. After that, the filtrates were concentrated at 37 °C under low atmospheric pressure, using a rotary evaporator to remove all solvents and obtain dried extracts. The final dry weights were used to calculate extraction yields (%). All extracts were kept in a refrigerator at 4 °C for experimental analyses.

### 3.2. Reagents and Bacterial Strains

Standard references, including quercetin (≥95%), rutin hydrate (≥94%), gallic acid (≥97%), and dimethyl sulfoxide (DMSO), were purchased from Sigma-Aldrich (St. Louis, MO, USA). 1,1-Diphenyl-2-picrylhydrazyl (DPPH) and 2,2′-Azino-bis (3-ethylbenzo thiazoline-6-sulfonic acid) (ABTS) were purchased from WAKO Pure Chemical (Osaka, Japan). Trolox was purchased from Calbiochem (San Diego, CA, USA). HPLC-grade methanol, phosphoric acid, and other compounds at analytical levels, as well as media used for bacterial growth, such as Muller Hinton agar or broth, were purchased from Merck (Darmstadt, Germany). Tested bacteria, including three gram (+) strains: *B. cereus* ATCC 11778, *B. subtilis* ATCC 6633, and *S. aureus* ATCC 25923; and three gram (−) strains: *E. coli* ATCC 25922, *P. aeruginosa* ATCC 9027, and *S*. typhimurium ATCC 13311, were purchased from the American Type Culture Collection (ATCC, Rockville, MD, USA).

### 3.3. HPLC Analysis of Rutin and Quercetin

Analysis of rutin and quercetin was performed using HPLC techniques, following the “General instructions for the determination of flavonoid content by HPLC method”, established by the National Institute for Food Control and accredited by the Vietnam Standards and Quality Institute (Code NIFC.05.M.235. Documentary number: 894.2020/QÐ-VPCNCL, issued 2020) [110], with some modifications. Briefly, the system consisted of an Agilent C18 (250 mm × 4.6 mm × 5 μm) column, which was connected to a 1260 Agilent HPLC (Agilent Technologies, Palo Alto, CA, USA) and equipped with a UV detector. The mobile phases were A: 0.1% acetic phosphoric in double-deionized water and B: methanol. The gradient conditions were as follows: solvent B: 0 min, 10%; 1 min, 10%; 5 min, 50%; 13 min, 70%; 16–20 min, 10%. Other chromatographic conditions were as follows: flow rate: 1 mL/min, column temperature: 30 °C, and run time: 20 min. The wavelength of detection was 360 nm. Methanol was used as a diluent for rutin, quercetin references, and extracts. Solutions were then passed through a 0.45 µm filter (Whatman plc., Buckinghamshire, United Kingdom) before being subjected to HPLC analysis. The contents of rutin or quercetin in samples were calculated by comparing the sample peak areas (% fluorescence) with those in the standard curves for rutin or quercetin references. HPLC analysis of extracts was performed in triplicate.

### 3.4. Evaluation of Antibacterial Effects of the Extracts

The effects of extracts on bacteria were evaluated through broth dilution and agar well diffusion methods, following our previous study [91] with some modifications. To observe dose-dependent effects, 10% dimethyl sulfoxide (DMSO) was used to dilute extracts and purified compounds, such as rutin or quercetin, to obtain serially tested concentrations. The broth dilution method was performed to determine the minimum inhibitory concentration (MIC) values following the methods of the Clinical and Laboratory Standards Institute [111], with some modifications to adjust to the necessary conditions for testing plant materials [91]. Tested solutions were mixed with Muller Hinton broth in a 96-well microplate to produce serial dilutions ranging from 4000 μg/mL to 3.91 μg/mL. The final bacterial concentration was adjusted to 5 × 10^5^ cfu/mL. All bacteria were incubated at 37 °C for 24 h. The lowest concentration that completely inhibited the visible growth of a microorganism was recorded as the MIC [111]. In addition, 10% DMSO served as a negative control, and kanamycin was used as a positive and quality control. Its MIC against *E. coli* ATCC 25922 was determined to be 2 μg/mL, which was within the acceptable limits (from 1–4 μg/mL) established by the Clinical and Laboratory Standards Institute [111]. The agar well diffusion method was performed following Nguyen et al. [91]. Briefly, a Muller Hinton agar plate was inoculated with bacteria at a final concentration of 10^6^ cfu/mL, and a hole with a diameter of 10 mm was punched aseptically with a cork borer. Then, 100 μL of tested materials, including extracts and isolated compounds, at established concentrations, were added into the well. Agar plates were incubated at 37 °C for 24 h. Inhibition zones, formed by the diffusion of samples in agar, were identified as the regions of petri plates surrounding the wells that did not have visible bacterial growth. Inhibitory diameters (excluding 10 mm of well diameters) were then measured to assess the magnitude of bacterial growth suppression [91]. In these experiments, extracts were examined at concentrations starting at 30 mg/mL, while those of rutin and quercetin were respectively at 25 mg/mL and 3 mg/mL. The 10% DMSO induced no inhibition and was applied as a negative control. Experiments were performed in triplicate.

### 3.5. Determination of Total Polyphenols, Total Flavonoids, and Antioxidant Activities

Total polyphenol contents were measured following the method of Suda et al. [112], with some modifications. For the control, 0.2 mL of extracts diluted with DMSO (1 mg/mL) was added to 1 mL of Folin Ciocalteu. This mixture was allowed to stand at room temperature for 3 min. Then, 1 mL of 10% Na_2_CO_3_ and 5 mL of distilled water were added. After 1 h of incubation, the absorbance was measured using a spectrophotometer (ERMA model AE-450, Saitama, Japan) at 750 nm. For the blank, a sample without Folin Ciocalteu reagent was taken along as well. All experiments were performed in triplicate, and gallic acid was used as a standard substance. Gallic acid reference at different concentrations (from 2 to 30 µg/mL) was applied to establish a standard calibration curve, and total polyphenol contents were expressed as mg gallic acid equivalent per g (GAE mg/g) weight of dried extract. Experiments in the measurement of total polyphenol contents were performed in triplicate.

The total flavonoid contents were determined according to the aluminum chloride method [113]. Briefly, 0.5 mL of extracts diluted with DMSO (25 µg/mL) was mixed with 300 µL of NaNO_2_ 5%, and the test tube was allowed to stand at room temperature for 5 min. After that, 300 µL of AlCl_3_ 10% and 2 mL of NaOH 1M were added. Finally, 1.9 mL of distilled water was added to the reaction mixture, and the absorbance of the solution was measured at 510 nm. 

The antioxidant activities of the extracts were evaluated through DPPH and ABTS radical scavenging activity.

DPPH scavenging activity was measured with DPPH, according to the procedure described by Masuda et al. [114], and with some modifications. DPPH solution was made by mixing 0.1 g of DPPH powder with 50 mL of ethanol at 96%. Briefly, to 0.2 mL of extracts (from 6 to 22 µg/mL) or isolated compounds diluted with DMSO, 0.1 mL of DPPH solution and 4.8 mL of DMSO were added. The absorbance was determined using the spectrophotometer at a 515 nm wavelength with a blank containing only the sample and solvent. For the control absorbance measurement, 0.1 mL of DPPH solution was added to 4.9 mL of DMSO solvent. The result was expressed as a percentage of antioxidant activity, calculated using the equation:

$$\mathrm{DPPH}\;\mathrm{radical}\;\mathrm{scavenging}\;\left(\%\right)=[\mathrm{Ac}-\left(\mathrm{As}-\mathrm{Ab}\right)]/\mathrm{Ac}\;\times 100$$

where Ac (A control) is the absorbance of DMSO and DPPH solution; As (A sample) is the absorbance of extracts and DPPH solution; and Ab (A blank) is the absorbance of extracts and DMSO.

ABTS scavenging activity was measured with ABTS, according to the procedure described by Wetwitayaklung et al. [115], with some modifications. To produce an ABTS^+^ radical solution, 7 mM of ABTS in water was reacted equally with 4.9 mM of potassium persulfate (K_2_S_2_O_8_) in water. This mixture was allowed to stand in the dark at room temperature for 12 to 16 h. The working solution was prepared by diluting the ABTS^+^ radical solution with water until the absorbance was 0.70 ± 0.02 at 734 nm using a spectrophotometer. In the assay, 0.2 mL of extracts (from 2 to 16 µg/mL) or isolated compounds diluted with DMSO was mixed with 3.8 mL of ABTS^+^ radical solution. The absorbance at 734 nm was determined after 4 min. For each sample, a blank was prepared with 3.8 mL of water, instead of the ABTS^+^ radical solution. All samples were evaluated in triplicate. The % ABTS radical scavenging activity was calculated using the equation:

$$\mathrm{ABTS}\;\mathrm{radical}\;\mathrm{scavenging}\;\left(\%\right)=\left[\mathrm{Ac}-\left(\mathrm{As}-\mathrm{Ab}\right)/\mathrm{Ac}\right]\;\times 100$$

where Ac (A control) is the absorbance of DMSO and ABTS solution; As (A sample) is the absorbance of extracts and ABTS solution; and Ab (A blank) is the absorbance of extracts and DMSO.

All experiments measuring DPPH and ABTS scavenging activities were performed in triplicate. Trolox (from 1 to 6 µg/mL) and gallic acid (from 1 to 4 µg/mL) were used as reference substances. The plotted graphs between % scavenging and concentration were used to calculate the half-maximal inhibitory concentration (IC_50_) values, the concentrations required to inhibit 50% of DPPH or ABTS radical scavenging.

### 3.6. Statistical Analysis

Results are expressed as means ± standard deviation (SD). Statistical analyses were performed by an unpaired *t*-test or Tukey’s test after one-way analysis of variance (one-way ANOVA). Significance was established when the probability level was equal to or less than 5%.

## 4. Conclusions

For the first time, our study has demonstrated that roasting under appropriate conditions can significantly enhance the antibacterial properties of SJ buds, providing scientific evidence to support the traditional knowledge of using this method for preparing SJ as herbal medicine. Furthermore, roasting can also improve other biological values, such as polyphenol content and antioxidant activities, which would properly increase the beneficial effects of this material. The enhancements in both antibacterial and antioxidant activities are mediated via changes in phytochemical constituents as a result of heating, including the conversion of rutin to quercetin, which has stronger potencies in both effects. The optimal roasting condition that can simultaneously potentiate antibacterial, total phenolic, and antioxidant properties was identified as dark yellow-roasting, suggesting that this method should be employed for SJ buds used in therapies associated with these medicinal functions. The results of our study serve as the basis for the proper roasting conditions for SJ buds to optimize their antibacterial properties. However, further research is still necessary to confirm the advantages under in vivo conditions and to widen the application of roasted SJ in practice. In addition, it is also worth investigating the role of roasting in other pharmacological properties, as proper treatments might significantly potentiate the therapeutic functions and exploit the potential of SJ buds.

## Figures and Tables

**Table 1:** Contents of rutin and quercetin in non-roasted, yellow-roasted, dark yellow-roasted, scorched-roasted, and charred-roasted samples of *Sophora japonica* bud materials.

Sophora Japonica Bud Material	Extraction Yield (%)	Concentration (mg/g Dried Extract)		Concentration (mg/g Dried Powder)	
		Rutin	Quercetin	Rutin	Quercetin
Non-roasted	32.54 ± 0.05	784.73 ^a^ ± 4.03	43.76 ^c^ ± 1.05	255.04 ^a^ ± 1.31	14.22 ^c^ ± 0.34
Yellow-roasted	32.50 ± 0.16	782.08 ^a^ ± 5.26	44.22 ^c^ ± 0.95	254.17 ^a^ ± 1.71	14.37 ^c^ ± 0.31
Dark yellow-roasted	32.51 ± 0.12	683.70 ^b^ ± 4.38	86.96 ^a^ ± 0.71	222.20 ^b^ ± 1.42	28.26 ^a^ ± 0.23
Scorched-roasted	32.53 ± 0.10	631.56 ^c^ ± 3.27	58.75 ^b^ ± 1.20	205.26 ^c^ ± 1.06	19.09 ^b^ ± 0.39
Charred-roasted	32.52 ± 0.09	387.23 ^d^ ± 4.33	21.17 ^d^ ± 0.34	125.85 ^d^ ± 1.41	6.88 ^d^ ± 0.11

Results are expressed as mean ± standard deviation (SD) of three tests. Values with different superscripts indicate a significant difference (*p* < 0.05) compared with other values of the same column, assessed by one-way ANOVA followed by Tukey’s post hoc test.

**Table 2:** Minimum inhibitory concentration (µg/mL) of methanol extracts of *Sophora japonica* bud materials, quercetin, and rutin against bacteria.

Tested Material	Gram (+)			Gram (−)		
	*Bacillus cereus*	*Bacillus subtilis*	*Staphylococcus aureus*	*Escherichia coli*	*Salmonella typhimurium*	*Pseudomonas aeruginosa*
Non-roasted bud	125	125	62.5	500	1000	1000
Yellow-roasted bud	125	125	62.5	500	1000	1000
Dark yellow-roasted bud	31.3	31.3	15.6	125	250	250
Scorched-roasted bud	62.5	62.5	31.3	250	250	250
Charred-roasted bud	250	250	125	500	2000	2000
Quercetin	500	500	250	1000	2000	2000
Rutin	4000	4000	2000	4000	4000	4000

**Table 3:** Inhibitory zones (mm) induced by methanol extracts of *Sophora japonica* buds and purified rutin or quercetin on bacteria.

Bacterium		Material	Concentration (mg/mL)			
Gram (+)	*Bacillus cereus* ATCC 11778	Bud extract	30 mg/mL	15 mg/mL	7.5 mg/mL	3.8 mg/mL
		Non-roasted	5.21 ^c^ ± 0.22	2.26 ^c^ ± 0.22	-	-
		Yellow-roasted	5.18 ^c^ ± 0.30	2.29 ^c^ ± 0.18	-	-
		Dark yellow-roasted	**15.52 ^a^ ± 0.61**	9.26 ^a^ ± 0.39	6.08 ± 0.17	2.17 ± 0.25
		Scorched-roasted	10.63 ^b^ ± 0.28	7.41 ^b^ ± 0.37	-	-
		Rutin	25 mg/mL	12.5 mg/mL	6.3 mg/mL	3.1 mg/mL
			4.67 ± 0.50	-	-	-
		Quercetin	3 mg/mL	1.5 mg/mL	0.8 mg/mL	0.4 mg/mL
			12.59 ± 1.01	8.02 ± 0.11	-	-
	*Bacillus subtilis* ATCC 6633	Bud extract	30 mg/mL	15 mg/mL	7.5 mg/mL	3.8 mg/mL
		Non-roasted	5.39 ^c^ ± 0.45	2.24 ^c^ ± 0.29	-	-
		Yellow-roasted	5.36 ^c^ ± 0.55	2.27 ^c^ ± 0.27	-	-
		Dark yellow-roasted	**15.63 ^a^ ± 0.76**	9.66 ^a^ ± 0.69	5.77 ± 0.52	2.48 ± 0.48
		Scorched-roasted	11.40 ^b^ ± 0.67	6.62 ^b^ ± 0.51	-	-
		Rutin	25 mg/mL	12.5 mg/mL	6.3 mg/mL	3.1 mg/mL
			3.80 ± 0.27	-	-	-
		Quercetin	3 mg/mL	1.5 mg/mL	0.8 mg/mL	0.4 mg/mL
			13.47 ± 0.65	9.00 ± 0.19	-	-
	*Staphylococcus aureus* ATCC 25923	Bud extract	30 mg/mL	15 mg/mL	7.5 mg/mL	3.8 mg/mL
		Non-roasted	9.04 ^c^ ± 0.05	5.69 ^c^ ± 0.71	2.78 ^c^ ± 0.46	-
		Yellow-roasted	9.06 ^c^ ± 0.17	5.72 ^c^ ± 0.70	2.69 ^c^ ± 0.34	-
		Dark yellow-roasted	**22.1 ^a^ ± 1.25**	16.8 ^a^ ± 0.74	10.7 ^a^ ± 0.41	7.07 ± 0.59
		Scorched-roasted	13.8 ^b^ ± 0.42	9.66 ^b^ ± 0.60	4.60 ^b^ ± 1.50	-
		Rutin	25 mg/mL	12.5 mg/mL	6.3 mg/mL	3.1 mg/mL
			5.41 ± 0.46	-	-	-
		Quercetin	3 mg/mL	1.5 mg/mL	0.8 mg/mL	0.4 mg/mL
			15.68 ± 1.02	10.73 ± 1.39	-	-
Gram (−)	*Escherichia coli* ATCC 25922	Bud extract	30 mg/mL	15 mg/mL	7.5 mg/mL	3.8 mg/mL
		Non-roasted	**-**	-	-	-
		Yellow-roasted	**-**	-	-	-
		Dark yellow-roasted	12.63 * ± 1.08	9.19 * ± 0.27	4.14 * ± 0.28	-
		Scorched-roasted	9.53 ± 0.40	5.07 ± 0.24	2.08 ± 0.18	-
		Quercetin	3 mg/mL	1.5 mg/mL	0.8 mg/mL	0.4 mg/mL
			10.43 ± 0.82	8.07 ± 0.23	-	-
	*Salmonella* typhimurium ATCC 13311	Bud extract	30 mg/mL	15 mg/mL	7.5 mg/mL	3.8 mg/mL
		Non-roasted	**-**	-	-	-
		Yellow-roasted	**-**	-	-	-
		Dark yellow-roasted	8.10 * ± 0.22	5.38 * ± 0.45	2.82 ± 0.27	-
		Scorched-roasted	4.70 ± 0.34	2.06 ± 0.19	-	-
		Quercetin	3 mg/mL	1.5 mg/mL	0.8 mg/mL	0.4 mg/mL
			8.75 ± 0.43	6.08 ± 0.13	-	-
	*Pseudomonas aeruginosa* ATCC 9027	Bud extract	30 mg/mL	15 mg/mL	7.5 mg/mL	3.8 mg/mL
		Non-roasted	**-**	-	-	-
		Yellow-roasted	**-**	-	-	-
		Dark yellow-roasted	9.04 * ± 0.23	6.07 * ± 0.17	2.56 ± 0.42	-
		Scorched-roasted	5.10 ± 0.23	1.52 ± 0.51	-	-
		Quercetin	3 mg/mL	1.5 mg/mL	0.8 mg/mL	0.4 mg/mL
			7.75 ± 0.35	5.37 ± 0.45	-	-

Results are expressed as means ± standard deviation (SD) of three tests.—means no inhibition. Charred-roasted materials showed no inhibitory zones at all tested concentrations and were not shown. Values with different superscript letters indicate significant differences (*p* < 0.05) compared with other values from different extracts of similar concentrations on the same bacterium, assessed by one-way ANOVA followed by post hoc Tukey’s test. * indicates significant difference (*p* < 0.05) between the inhibitory zones induced by Dark yellow-roasted extract vs. that of Scorched-roasted extract at similar concentrations and for gram (−) bacteria, assessed by unpaired *t*-test. Bold letters indicate the largest values of inhibitory zones induced by different tested extracts on the same bacterium.

**Table 4:** IC_50_ of *Sophora japonica* bud extracts and isolated compounds assessed using DPPH and ABTS assays.

Tested Material	IC_50_ (µg/mL)	
	DPPH	ABTS
**Standard reference**		
Trolox	3.75 ± 0.03	2.91 ± 0.04
Gallic acid	2.75 ± 0.06	1.97 ± 0.02
***Sophora japonica* extract**		
Non-roasted extract	14.91 ^c^ ± 0.33	8.22 ^c^ ± 0.11
Yellow-roasted extract	15.60 ^c^ ± 0.10	8.24 ^c^ ± 0.11
Dark yellow-roasted extract	9.31 ^a^ ± 0.10	5.25 ^a^ ± 0.05
Scorched-roasted extract	12.77 ^b^ ± 0.15	6.66 ^b^ ± 0.04
Charred-roasted extract	20.80 ^d^ ± 0.26	9.70 ^d^ ± 0.14
**Isolated compound**		
Quercetin	2.93 * ± 0.02	2.04 * ± 0.01
Rutin	9.65 ± 0.06	4.54 ± 0.02

Results are expressed as means ± standard deviation (SD) of three tests. Values with different superscript letters indicate significant differences (*p* < 0.05) compared with other values from different extracts, assessed by one-way ANOVA followed by post hoc Tukey’s test. * indicates significant difference (*p* < 0.05) between the values of quercetin and rutin, assessed by an unpaired *t*-test.

## Data Availability

All data are shown in the manuscript and Supplementary Materials Data S1.

## Supplementary Materials

The following supporting information can be downloaded at: https://www.mdpi.com/article/10.3390/plants13233337/s1.

## Abbreviations

SJ: *Sophora* japonica; ABTS: 2,2′-Azino-bis (3-ethylbenzo thiazoline-6-sulfonic acid); DPPH: 1,1-Diphenyl-2-picrylhydrazyl; DMSO: dimethyl sulfoxide; gram-positive: gram (+); gram-negative: gram (-); MIC: minimum inhibitory concentration; IC_50_: half maximal inhibitory concentration; RT: retention time; *B. cereus*: *Bacillus cereus; B. subtilis*: *Bacillus subtilis; E. coli*: *Escherichia coli*; *P. aeruginosa*: *Pseudomonas aeruginosa*; *S. aureus*: *Staphylococcus aureus*; *S*. *typhimurium*: *Salmonella enterica* subsp. *enterica* serovar Typhimurium.
